# PADG‐Pred: Exploring Ensemble Approaches for Identifying Parkinson's Disease Associated Biomarkers Using Genomic Sequences Analysis

**DOI:** 10.1049/syb2.70006

**Published:** 2025-03-15

**Authors:** Ayesha Karim, Tamim Alkhalifah, Fahad Alturise, Yaser Daanial Khan

**Affiliations:** ^1^ Department of Computer Science School of Systems and Technology University of Management and Technology Lahore Pakistan; ^2^ Department of Computer Engineering, College of Computer Qassim University Buraydah Saudi Arabia; ^3^ Department of Cybersecurity College of Computer Qassim University Buraydah Saudi Arabia

**Keywords:** artificial intelligence, data analysis, feature extraction, genomics, machine learning, medical computing, pattern classification

## Abstract

Parkinson's disease (PD), a degenerative disorder affecting the nervous system, manifests as unbalanced movements, stiffness, tremors, and coordination difficulties. Its cause, believed to involve genetic and environmental factors, underscores the critical need for prompt diagnosis and intervention to enhance treatment effectiveness. Despite the array of available diagnostics, their reliability remains a challenge. In this study, an innovative predictor PADG‐Pred is proposed for the identification of Parkinson's associated biomarkers, utilising a genomic profile. In this study, a novel predictor, PADG‐Pred, which not only identifies Parkinson's associated biomarkers through genomic profiling but also uniquely integrates multiple statistical feature extraction techniques with ensemble‐based classification frameworks, thereby providing a more robust and interpretable decision‐making process than existing tools. The processed dataset was utilised for feature extraction through multiple statistical moments and it is further involved in extensive training of the model using diverse classification techniques, encompassing Ensemble methods; XGBoost, Random Forest, Light Gradient Boosting Machine, Bagging, ExtraTrees, and Stacking. State‐of‐the‐art validation procedures are applied, assessing key metrics such as specificity, accuracy, sensitivity/recall, and Mathew's correlation coefficient. The outcomes demonstrate the outstanding performance of PADG‐RF, showcasing accuracy metrics consistently achieving ∼91% for the independent set, ∼94% for 5‐fold, and ∼96% for 10‐fold in cross‐validation.

## Graphical abstract

This study introduces PADG‐Pred, a novel computational model leveraging genomic data to identify Parkinson's disease biomarkers with high accuracy, utilising advanced ensemble classification techniques and rigorous cross‐validation to enhance early diagnostic capabilities.

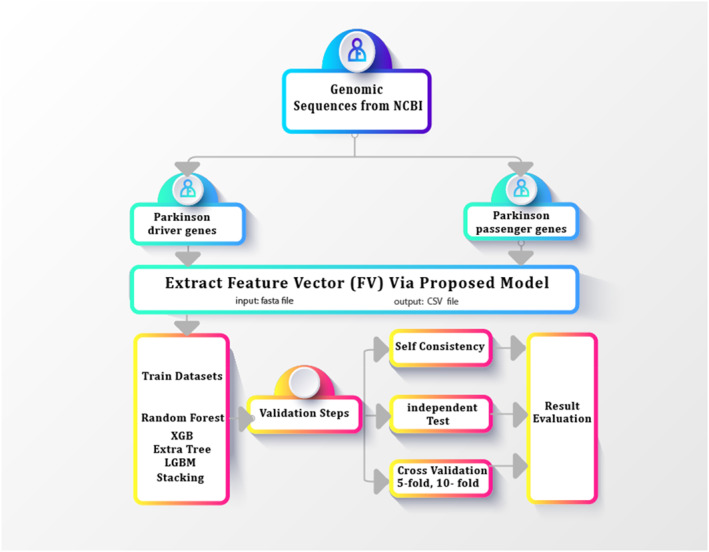

## 1 Introduction

Parkinson's disease (PD) is a progressive neurodegenerative disorder characterised by the loss of dopamine‐producing neurons in the substantia nigra [1, 2, 3, 4]. The genetic basis of PD is complex, with both rare and common genetic variants contributing to disease susceptibility. Studies on the genetic basis of PD began in the late 1980s, with the discovery of mutations in the alpha‐synuclein gene (SNCA) in families with autosomal dominant PD. Subsequently, several other genes have been identified that are associated with PD, including parkin (PARK2), PINK1, (PARK6), DJ‐1 (PARK7), GBA, LRRK2 (PARK8), and SNCA [5, 6, 7, 8, 9]. These genes play important roles in the regulation of dopamine neurotransmission [10, 11, 12], the ubiquitin‐proteasome system [13, 14, 15], and the autophagic‐lysosomal pathway [16, 17], all of which are thought to be involved in the pathogenesis of PD. Common genetic variations in several genes have also been associated with PD susceptibility, including the SNCA, LRRK2, GBA, and MAPT genes [5, 18, 19, 20]. Studies have also identified genetic risk factors in several genomic regions, including the major histocompatibility complex (MHC) [18, 21, 22, 23] region on chromosome 6.

Genome‐wide association studies (GWAS) and exome‐wide association studies (EWAS) have facilitated several studies on PD, its risk factors, reasons, and treatments [24, 25, 26]. The motivation for this study is to bridge the gap between genetic research and practical diagnostic tools, providing clinicians with a reliable computational model for identifying Parkinson's disease biomarkers. Various computational techniques have been developed to diagnose Parkinson's disease at an early stage. The use of artificial neural networks (ANN) stands out as it simulates neuronal behaviour for symptom identification. Additionally, many systems including expert systems (ES), and fuzzy logic (FL) used to recognise patterns associated with the disease. The neuroimaging technologies single photon emission computed tomography (SPECT), transcranial sonography, magnetic resonance imaging (MRI), positron emission tomography (PET), and functional magnetic resonance imaging (FMRI) are widely used for PD diagnosis [27, 28]. However, these imaging techniques have limitations; for instance, SPECT and PET only become effective after 80% of neurons are damaged, and MRI, despite advancements is still limited to disease characterisation. Additionally, deep transfer learning through handwriting datasets has achieved success in Parkinson's identification [29, 30]. Audio signal processing also gained attention in the detection of Parkinson's using machine learning techniques; naïve Bayes classifier, KNN, SVM, and multilayer perceptron (MLP), where CNN showed promising results [31, 32]. The goal of this study is to propose PADG‐Pred, a computational model specifically designed to identify genetic biomarkers of Parkinson's disease from genomic data. This model is significant because it provides a practical use case: aiding in early and precise diagnosis, which is essential for improving treatment strategies. The multifaceted computational approaches [33, 34] devised aim to uncover genetic complexities linked with Parkinson's disease. In ref. [35], challenges and limitations of computational methods for the identification of genetic variation associated with Parkinson's disease are covered. In refs. [36, 37], machine learning techniques including support vector machine (SVM), random forest (RF), and neural networks NN are involved in the identification of genetic variation and future research.

The paper provides the details of computational methodologies that have been analysed for the prediction of Parkinson's disease using genetic sequences available through genome‐wide association studies (GWAS). Our approach goes beyond simple data processing. In this study, innovative predictor PADG‐Pred ‘PADG‐Pred: Parkinson's Associated or Driver Gene Predictor’ is proposed for the identification of Parkinson's associated biomarkers, utilising a genomic profile. We begin by (1) developing a significant benchmark dataset for rigorous testing and training. The next step involves (2) applying robust numerical equations to accurately reflect target variations. (3) An algorithm is then formulated for effective prediction, followed by (4) training the model with gathered datasets. Finally, (5) we evaluate the model to ensure credibility and high accuracy. This process aims to generate meaningful and clinically relevant prediction of Parkinson's associated biomarkers that can assist practitioners in diagnosing and treating Parkinson's disease.

## 2 Materials and Methods

**FIGURE 1:**
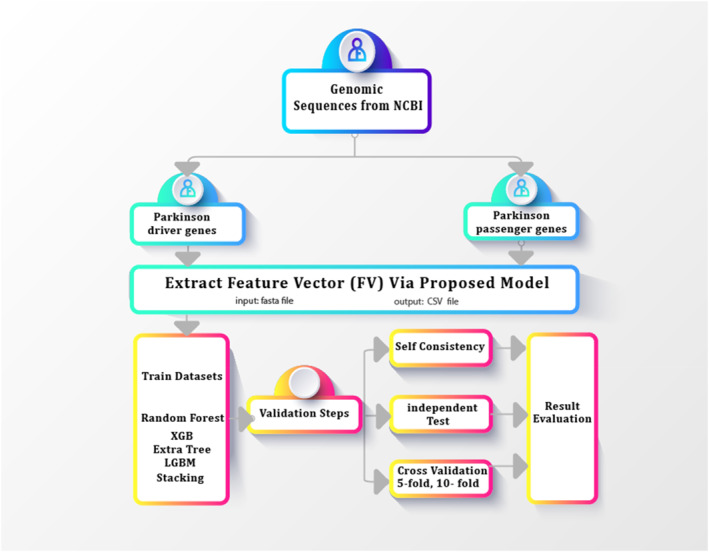
Architecture flow illustrating the procedure of training, testing, and evaluation.

This research process is outlined in several key steps as illustrated in Figure 1. First, we begin with the collection of relevant datasets from the NCBI database [38]. These datasets contain critical genetic information related to Parkinson's disease, serving as the foundational data for our analysis. Second, the data processing stage involves rigorous cleaning and standardisation techniques. Irrelevant and incomplete data points are removed to ensure the dataset's integrity and relevance. Following this, data standardisation is performed to adjust the values for consistency, and normalisation scales the data to a uniform range, which enhances the performance and stability of the predictive models. Third, the feature encoding process transforms the cleaned and processed dataset into structured feature vectors. This step employs a variety of numerical and statistical techniques to extract meaningful patterns from the genetic sequences. These feature vectors encapsulate the essential characteristics of the genetic data, making them suitable inputs for machine learning algorithms. Fourth, the model training phase is a critical component of the methodology. We train multiple machine learning classifiers, including RF, XGBoost, LGBM, and Stacking ensembles. These classifiers are selected for their ability to handle complex data patterns and are extensively trained on the extracted feature vectors to build a robust predictive model capable of accurately identifying genetic markers of Parkinson's disease. Finally, the evaluation phase is conducted to rigorously assess the performance and reliability of the trained models. This involves using an independent test set to validate the model's generalisability and performing cross‐validation with both 5‐fold and 10‐fold methods to ensure consistency and reduce overfitting. Self‐consistency checks are also employed to confirm the stability of the predictions. These comprehensive evaluation techniques provide a thorough assessment of the model's predictive capabilities.

A comprehensive detail on usage, along with a description of the computational processes is discussed in this section as shown in Figure 1.

The input data for the proposed framework initially consists of FASTA files. From these sequences, feature vectors are extracted and then saved in an Excel file. This Excel file serves as the input for Python‐based machine learning algorithms, ultimately generating the final predictive results. The file types and their transitions are also reflected in Figure 1.

The study utilised various Python libraries to streamline data processing and model development. Pandas and NumPy were employed for data cleaning, standardisation, and efficient data manipulation. For machine learning, Scikit‐Learn, Random Forest, Extra Tree, XGBoost, and LightGBM were used to implement and train diverse classifiers, whereas Matplotlib and Seaborn facilitated data visualisation and result analysis.

### 2.1 Benchmark Dataset Collection and Its Preprocessing

Benchmark datasets, used as reference points for evaluating model performance, are typically built from well‐defined experimental samples. These unambiguous samples serve a dual purpose: training machine learning models and testing their accuracy. To ensure the effectiveness of the benchmark, the dataset must be diverse, accurate, and relevant to the task at hand. In this study, the benchmark dataset was sourced from the latest publicly available version on (https://www.ncbi.nlm.nih.gov/) [38]. This dataset lists the mutations in multi‐organism genes, with the majority being passenger mutations that are not associated with causing Parkinson's. The foundational steps involved selecting and mapping a benchmark dataset from NCBI. Each gene was verified against current literature to ensure relevance to Parkinson's disease. Only experimentally validated Parkinson‐related driver genes with comprehensive sequence data were included, while genes unassociated with the disorder were excluded. This curated dataset then formed the basis for feature vector creation, enabling the extraction of critical genetic patterns for training predictive models. There are 1815 PD‐causing mutations, referred to as Parkinson's driver genes. Subsequently, CD‐HIT [39] clustering was implemented at a threshold of 0.70 after the cleaning of the dataset. This strategic approach not only facilitated the identification of symmetrical genes but also significantly mitigated redundancy within the dataset.

In this study, the benchmark dataset is represented as P and is defined as follows:

(1) $$P=P^{+}{UP}^{-}$$

**FIGURE 2:**
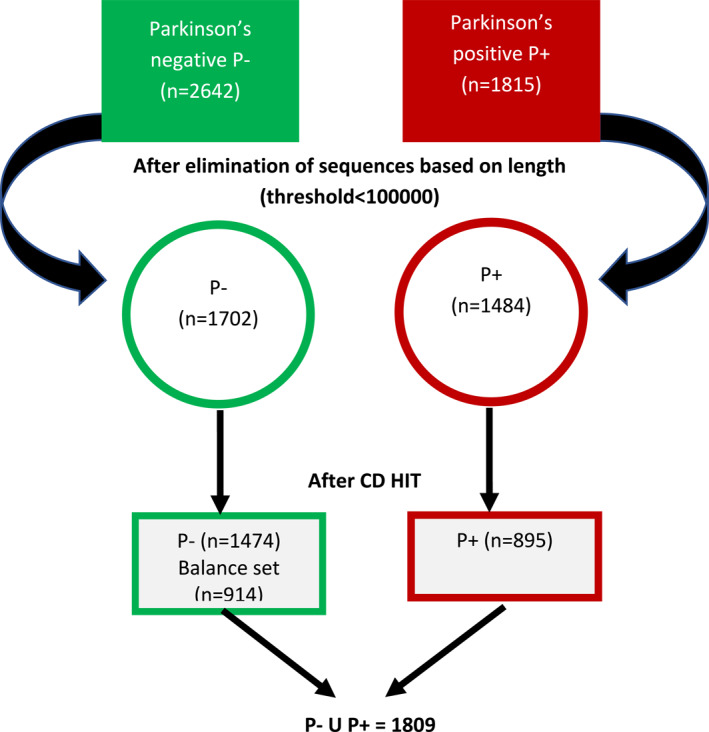
Data processing on extracted dataset.

Careful preparation was done to create this database, consisting of 895 positive multi‐organism gene sequences (*P* ^+^) and 914 negative samples selected from a large collection of passenger genes (*P* ^−^) after applying data processing and CD‐Hit. The negative gene sequences (*P* ^−^) were selected as shown in Figure 2.

Expressing the Sample, a DNA sequence can be represented as given below:

(2) $$S=\rho_{1},\rho_{2},\rho_{3},\text{…},\rho_{i},\text{…},\rho_{n}$$

Where at each position, the nucleotide is indicated by the following:

(3) ρ∈{A(adenine),C(cytosine),G(guanine),T(thymine)}

and ∈ denotes the membership within the set, the meaning of ‘member of’.

**FIGURE 3:**
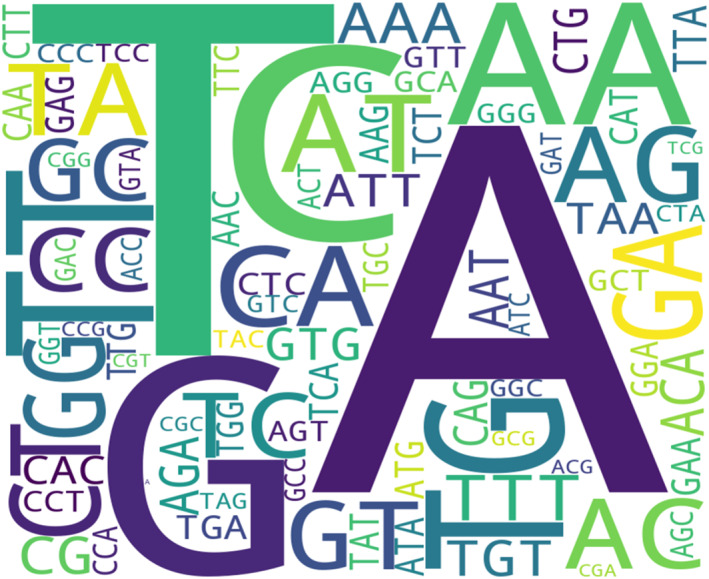
Word cloud of Parkinson's disease‐related dataset.

Figure 3 presents a word cloud visualisation depicting the frequency of DNA sequence elements *A*, *T*, C, and *G* within the dataset.

The progress in science has paved the way for exploring advances in biotechnology. However, transforming raw data into desired discrete quantifiable models remains a challenge, as it should be done without losing grouping characteristics and according to their grouping information. These models provide features and data that are crucial for intelligent target analysis. Machine algorithms such as ‘Light Gradient Boosting Machine(LGBM)’, ‘Random Forest (RF)’, and ‘XG Boost (XGB)’ are effective in evaluating vector information obtained from proteomic or genomic arrangements, as they are designed to handle vector inputs. To convert the entire sequence‐pattern data into a vector of a specific size in a discrete model, no information must be lost as it determines the sequence's features. The pseudo amino acid composition (PseAAC) was introduced to address the limitations of data gathered from proteins, and it has been widely used in computational proteomics through the computer application ‘PseAAC‐General.’ Its ability to deal with protein/peptide arrangements has led to the consideration of using it for dealing with DNA/RNA arrangements in computational genomics through the use of PseKNC (Pseudo K‐tuple Nucleotide Composition). Currently, transforming genomic profile into a well‐constructed mathematical encoding is possible through the representation *Z*′ of Equation (2) as follows:

(4) $$Z^{\prime }=\left[Z_{1}Z_{2}Z_{3}Z_{4}\text{…}Z_{u}\text{…}Z_{\alpha }\right]$$

where *Z*_*y*_ (*y* = 1, 2,…, α) represents an arbitrary‐numerical coefficient. The useful data extracted from the gene sequence is in the form of components of Equation (4), and the methodology [40, 41] used to extract these features will be discussed further.

### 2.2 Statistical Moments

In characterising the measurements and components of Equation (4), a factual approach is employed. To obtain fixed‐size data from genomic data, statistical moments are utilised. Each moment carries unique information that reveals the nature of the data. There has been extensive work done by mathematicians and analysts on the moments of various distributions. The feature set includes the Hahn, central, and raw moments of the genomic data. These moments play a critical role in the feature vector for the predictor. The moments that incorporate the scale and area of variance assist in distinguishing sequences that have different functions. Additionally, other moments that define asymmetry and the source of the data are valuable in constructing a classifier for a labelled dataset. Studies have shown that the properties of proteomic and genomic sequences are influenced by the relative positioning and composition of their bases. Thus, computational and mathematical models that are sensitive to the positioning of the nucleotide bases within genomic sequences are best suited to furnish the feature vector. This positioning is crucial in developing an effective and efficient feature set. One thing to note is that Hahn moments need data in a two‐dimensional format. So, we change the genetic sequences into a two‐dimensional form called *T*′, which has the same information as *T* but is organised differently. This helps us use Hahn moments effectively.

$$k=\sqrt{n}$$

and

(5) $$T^{\prime }=\left[\begin{array}{cccc} T_{11} & T_{12} & \text{⋯} & T_{1n}\\ T_{12} & T_{22} & \text{⋯} & T_{2n}\\ : & : & & :\\ T_{m1} & T_{m2} & \text{⋯} & T_{mn} \end{array}\right]$$

Additionally, we use mathematical calculations to create helpful information from our square matrix. This helps us make our set of features a specific size [42, 43, 44]. As mentioned before, we use three particular types of calculations in this study: Hahn, central, and raw estimates.

The raw moments of order *a*+b are calculated using Equation (6).

(6) $$D_{ab}=\sum_{p=1}^{n}\;\sum_{q=1}^{n}\;e^{a}f^{b}\delta_{pq}$$

The moments up to order 3, including *D*_00_, *D*_01_, *D*_10_, *D*_11_, *D*_12_, *D*_21_, *D*_30_, and *D*_03_, contain significant information. The central moments are calculated by first finding the centroid (*x*, *y*), which serves as the centre of the data and can be visualised as follows:

(7) $$V_{ab}=\sum_{g=1}^{n}\;\sum_{h=1}^{n}\;{\left(g-x\right)}^{a}{\left(h-y\right)}^{b}\delta_{gh}$$

The Hahn moments are calculated using Equation (8), where the square grid serves as the discrete input. The Hahn moments provide information on the symmetry of the data and are reversible, meaning the original data can be reconstructed in the future using these moments. This reversibility ensures the preservation of information in the original sequences and allows it to reach the predictor through the corresponding feature vector.

(8) $$h_{n}^{x,y}\left(p,K\right)={\left(K+V-1\right)}_{n}{\left(K-1\right)}_{n}\times \sum_{z=0}^{n}\;{\left(-1\right)}^{z}\frac{{\left(-n\right)}_{z}{\left(-p\right)}_{z}{\left(2K+x+y-n-1\right)}_{z}}{{\left(K+y-1\right)}_{z}{\left(K-1\right)}_{z}}\frac{1}{z!}$$

In Equation (8), the Pochhammer notation is utilised with the Gamma operator included. Typically, in Equation (8), the Hahn coefficient is normalised using the coefficient presented in Equation (9).

(9) $$H_{pq}=\sum_{j=0}^{K-1}\;\sum_{i=0}^{K-1}\;\delta_{pq}h_{q}^{a,b}{\left(j,K\right)h}_{q}^{a,b}\left(i,K\right),m,n=0,1,2,\text{…},K-1$$

### 2.3 Generation of Position Relative Incidence Matrix (PRIM)

The devised computational models are designed for certain applications, such as predicting the properties of genes and categorising them into fundamental groups to uncover their unique characteristics. The nucleotide bases also have a delicate quantisation of their role. The position of each base is crucial, as it determines its role. To capture the relative position of the nucleotide bases, a position relative incidence matrix [45, 46, 47] has been derived. The Aprim matrix, a 4 × 4 matrix defined in Equation (10), resulted in a total of 16 coefficients.

(10)

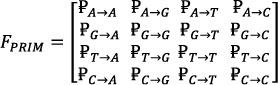

the relative position of each nucleotide (*A*, *C*, *T*, or *G*) to other nucleotides is represented by ₽i‐j. A 16 × 16 matrix, called *G*_*prim*_ Equation (10) is used to denote the Dinucleotide Composition (DNC), resulting in 16 unique combinations of nucleotides (e.g. *AA*, *AG*, *AT*…*CC*). Initially, this matrix produced 256 coefficients.
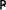


(11)

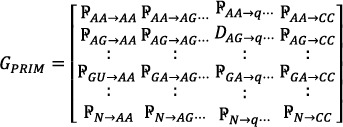




$H_{\mathrm{PRIM}}$ Equation (12) was formed to analyse 64 unique tri‐nucleotide base combinations (i.e. *AAA*, *AAG*, *AAT*, …, *CCG*, *CCT*, *CCC*). This matrix yielded a total of 4096 coefficients. Central, Hahn, and raw moments were then computed for F_*PRIM*_, $G_{\mathrm{PRIM}}$, and $H_{\mathrm{PRIM}}$, resulting in coefficients up to order 3.

(12)

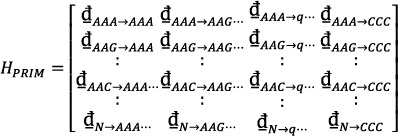




### 2.4 Formulation of Reverse Position Relative Indices Matrix (RPRIM)

The purpose of feature extraction is to identify hidden patterns within the genomic sequence, and various analyses are necessary to understand the behaviour of the gene. The experiment suggests that the reverse of a gene or protein sequence can provide valuable information. Therefore, the reverse position relative Indices matrix (RPRIM) is formed by reversing the original sequence and then computing the PRIM [48, 49, 50]. The matrix is created by first reversing the original sequence and then calculating the PRIM for the reversed sequence. In RPRIM, a random element, *R*_*n*→*m*_, represents the relative position of the *n*th base compared to the mth nucleotide base. The RPRIM matrix is expressed as follows:

(13)

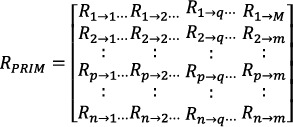




The Hahn, raw, and central moments are then calculated using the resulting matrix for consistency.

### 2.5 Frequency Matrices (FMs) Generation

The gene's characteristics are mostly determined by the arrangement and structure of nucleotides. The correlations between nucleotide base sequences are identified by PRIM and RPRIM matrices. The frequency vector contains the information regarding gene's composition. The FV summarises the information of genes based on frequency of occurrence of nucleotide, where each element represents the number of times a specific nucleotide appears. The vector as notated by the following:

(14) $$\alpha =\left\{{€}_{1}{,€}_{2,\text{…},}{€}_{n}\right\}$$

The €_*i*_ provides the total count of occurrences for the *i*th nucleotide base.

### 2.6 Accumulative Absolute Position Incidence Vector (AAPIV) Generation

The Accumulative Absolute Position Incidence Vector (AAPIV) is a feature extraction model that contains all relatable gene sequence composition [51, 52, 53]. The frequency of base sequence is determined by FV and Similar position of specific nucleotide base occurrence provided by AAPIV of length 4,16 and 64 as follows:

(15) $$V_{\mathrm{AAPIV}4}=\left\{\beta_{1}{,\beta_{2},\beta_{3},\beta }_{4}\right\}$$

(16) $$V_{\mathrm{AAPIV}16}=\left\{{\beta_{1}{,\beta_{2},\beta_{3},\beta }_{4},\text{…}..\beta_{15},\beta }_{16}\right\}$$

(17) $$V_{\mathrm{AAPIV}64}=\left\{{\beta_{1}{,\beta_{2},\beta_{3},\beta }_{4},\text{…}..\beta_{63},\beta }_{64}\right\}$$

For *i*th component

(18) $$\beta_{i}=\sum_{p=1}^{n}\;Bp$$

For a given nucleotide, the position of its occurrence is represented by β*p*. The sum of all the positions of the *i*th nucleotide can be found in an arbitrary element *i*.

### 2.7 Generation of Reverse Accumulative Absolute Position Incidence Vector (RAAPIV)

The patterns in the gene sequences are more viewable through the reverse sequence. The manipulation of AAPIV_4_, AAPIV_16_ & AAPIV_64_ for the reverse sequence is referred to as RAAPI V_4,_ RAAPI V_16,_ RAAPI V6_4_, and is represented as follows:

(19) $$V_{\text{RAAPIV}4}=\left\{{⋏}_{1}{,{⋏}_{2},{⋏}_{3},⋏}_{4}\right\}$$

(20) $$V_{\text{RAAPIV}16}=\left\{{{⋏}_{1}{,{⋏}_{2},{⋏}_{3},⋏}_{4},\text{…}..{⋏}_{15},⋏}_{16}\right\}$$

(21) $$V_{\text{RAAPIV}64}=\left\{{{⋏}_{1}{,{⋏}_{2},{⋏}_{3},⋏}_{4},\text{…}..{⋏}_{63},⋏}_{64}\right\}$$

In the reverse sequence, the sum of all the occurrences of the *i*th nucleotide is available in an arbitrary element *n*_*i*_ of the RAAPIV.

### 2.8 Feature Vector Formulation

The primary sequence in Equation (4) yields a fixed scale representation. The extensive matrices PRIM, RPRIM, and P′ are tweaked into a concise form, and moments are calculated by computing Hahn, raw, and central moments. These moments are then combined with RAAPIV, AAPIV, and FV to form a feature vector [53, 54]. The coefficients in feature vector correspond to a sequence of arbitrary size and length. For all samples, a holistic set of feature vectors is generated. The resulting set of 522 FV was finalized through a repetitive process of selection and fine‐tuning.

## 3 Ensemble Models Development

In this work, instead of a traditional machine learning algorithm the ensemble techniques are utilised to improve the classification accuracy of Parkinson's associated genes and non‐associated genes. The ensemble techniques are categorised into parallel and sequential models and both use different approaches for performance improvement. In this work stacking, boosting and bagging techniques have been applied to find Parkinson's disease‐related genes. These models are trained and validated using benchmark datasets of genomic profiles to enhance the reliability of results. The hyperparameter optimisation has been utilised in classification to address the possibility of overfitting and underfitting.

### 3.1 Random Forest (RF)

Random forests or random decision forests are collective learning methods for classification, regression, and other tasks by creating many decision trees at training time. This set of classifiers is represented in Equation (22) by the margin function qr(*A*, *Z*). It describes how the model uses a training set, randomly drawn from the distribution of a random vector (*Z*, *A*), to make predictions.

(22) $$\text{qr}\left(A,Z\right)=av_{k}I\left(h_{k}\left(A\right)=Z\right)-\max_{i\neq z}av_{k}I\left(h_{k}\left(A\right)=i\right)$$

Random Forest (RF) classifiers, utilising the sci‐kit‐learn library, rank genes based on their expression values within a DNA‐sequence sample set. This approach involved optimising hyperparameters such as maximum depth, maximum features, and minimum samples to achieve the best model performance. Hyperparameter tuning, significantly influenced the RF model's efficacy. In this study, the specific parameters, including a maximum depth of 25, automatic *max_features* selection, and *n_estimators* value of 50, through extensive experimentation have been established as shown in Table 1.

**TABLE 1:** Hyper‐parameter of machine learning Classifier's.

	Hyper parameter				
Classifier	*n_estimators*	*max_depth*	*oob_score*	*n_jobs*	*warm_start*
RandomForest	50	25	TRUE	−1	TRUE
LGBM	—	—	—	—	—
XGBClassifier	100	9	—	—	—
ExtraTrees	100	None	FALSE	None	FALSE
BaggingClassifier	10	—	FALSE	None	FALSE

### 3.2 XGBoost (Extreme Gradient Boosting)

This classifier is built through the supervised machine learning technique by tree boosting model and decision trees. The XGB is known for the extraction of patterns and features to train a predictor. XGB is based on decision trees having condition if/else (true/false) to determine the probability of correct decision by using minimum questions. The RF is used for regression and classification but the decision trees are built differently. The XGB model was hyper‐tuned by considering parameters such as *n_estimator* by 100 and depth of this boosting algorithm (*max_depth* = 9), and the *random_state*, which was set to ‘0.’

### 3.3 Light Gradient Boosting Machine Classifier

LGBMClassifier or Light Gradient Boosting Machine Classifier is a powerful machine learning algorithm known for its speed, accuracy, and efficiency in handling large datasets. It is a popular choice for classification tasks, where the goal is to categorise data into predefined classes. The LGBM model was hyper‐tuned by considering parameter *random_state = 42*.

### 3.4 Extra Tree

The extra tree classifier builds up many decision trees and the result is derived by taking the average or voting for the final decision. In this classifier, parameters are hyper‐tuned by considering *n_estimator* as 100, *obb_score* and *warm_start* as false.

### 3.5 Bagging

Bagging is treated as a grouping model to improve the efficiency and accuracy of machine learning models. The best part about this model is that it combines the multiple results of several models that are trained on different datasets and populates its final prediction. Model. This classifier was hyper‐tuned by making *n_estimators = 10*, *oob_score* and *warm_start = false* as shown in Table 1.

### 3.6 Stacking

Stacking is an efficient machine‐learning technique that involves combining several classifiers into one model by training meta‐models based on their predictions [54, 55]. In this study, the combination of *XGBClassifier, RandomForestClassifier, Logistic Regression*, and LGBM is employed for effective and reliable results. One of the best aspects of stacking is that it helps minimise the risk of overfitting.

## 4 Results & Discussion

It is critical to assess prediction algorithms' performance to establish their validity. Scientists have developed several quantitative metrics to assess prediction performance. These measurements, which will be used for model comparisons, are founded on tested procedures and practical experience. Computational predictor performance is usually assessed using four interconnected metrics. The accuracy metric, represented by Acc, gauges how accurate forecasts are overall. Additionally, specificity (*S*_*p*_), which gauges the prediction accuracy of negative samples, and sensitivity (*S*_*n*_), which represents the prediction accuracy of positive samples, were employed [56]. In cases where there is an imbalance between the positive and negative samples, Acc is assessed using the Mathew Correlation Coefficient (MCC), The metrics in 2013 [57] as follow:

(23) $$S_{p}=1-\frac{A^{\pm }}{A^{-}}0\leq S_{p}\leq 1$$

(24) $$S_{n}=1-\frac{A^{\pm }}{A^{-}}0\leq S_{n}\leq 1$$

(25) $$\text{Acc}=1-\frac{A_{-}^{+}+A_{+}^{-}}{A_{-}^{+}+A_{+}^{-}}0\leq \text{Acc}\leq 1$$

(26) $$\text{MCC}=\frac{1-\left(\frac{A^{\pm }}{A^{+}}+\frac{A^{\mp }}{A^{-}}\right)}{\sqrt{\left(1+\frac{{A^{\mp }-A}^{\pm }}{A^{+}}\right)\;\left(1+\frac{A^{\pm }-A^{\mp }}{A^{-}}\right)\;}}$$

The performance of prediction algorithms is crucial for their validation and is assessed using various quantitative metrics. Four main metrics are commonly used, including accuracy (Acc), sensitivity ($S_{n}$), specificity ($S_{p}$), and the Mathew's correlation coefficient (MCC). These metrics measure the performance of the predictor in predicting positive and negative samples correctly.

In the equation, $A^{+}$ signifies the definite number of Parkinson's ‐suspect genes, while $A^{\pm }$ is the number of Parkinson's ‐suspect genes as passenger genes. $A^{-}$ represents the actual passenger genes, and $A^{\mp }$ are the passenger genes predicted as Parkinson's ‐suspect genes. The sensitivity $S_{n}$ and specificity $S_{p}$ are maximised when every sample is correctly identified and no passenger gene is wrongly predicted. The sensitivity ($S_{n}$) and specificity ($S_{p}$) are maximised when no sample is wrongly predicted as passenger gene (i.e., $A^{\pm }=A^{\mp }=0$). The model is considered more accurate when Mathew's correlation coefficient and Accuracy are calculated as 1, indicating that no sample is wrongly predicted.

### 4.1 Self‐Consistency Validation

The self‐consistency validation is a commonly used method to assess the Acc of a predictor and is considered a basic test. The first step in this process is to create a set of feature vectors with both positive and negative gene samples, used to train the model.

After adequate training, the next step is to validate the model, with self‐consistency being the first test. This test involves using the same samples that were used for training to evaluate the predictor. A benchmark dataset is used to compare the performance of various classifiers, including Random forest, Bagging, LGBM, ET, and XGB using this validation method, as shown in Table 2.

**TABLE 2:** Results of self‐consistency experiment.

	Acc	Sensitivity/Recall	Specificity	MCC
Random forest	100	100	100	100
LGBM	100	100	100	100
XGB	100	100	100	100
Extra tree	100	100	100	100
Bagging	100	100	100	100
Stacking	98.62	99.45	97.77	97.0

**FIGURE 4:**
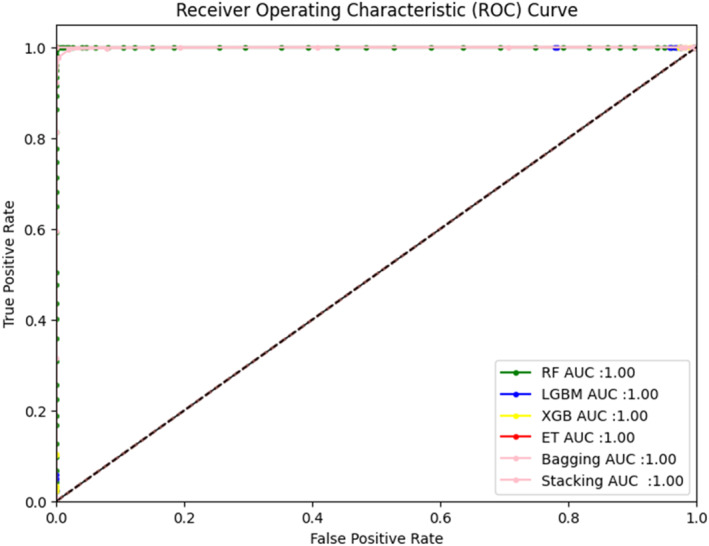
Results of self‐consistency ROC.

It has been observed that RF, XGB, ET, Bagging, and Stacking show exceptional performance in the graph in Figure 4. These results suggest that the predicted outcome aligns with the original computational method proposed.

In this study, self‐consistency validation uses the same dataset for both training and testing, providing performance metrics on the full dataset. To guard against overfitting, we also conduct independent testing, as well as 5‐fold and 10‐fold cross‐validation, splitting the data into 70% for training and 30% for testing. This ensures that the model is evaluated on unseen data, enhancing the robustness of our validation process.

### 4.2 Independent Dataset Test

The performance of prediction algorithms on unknown data sets can be determined through independent set tests. This involves dividing the data into two unequal parts, with the larger part being used for training the model and the smaller part for testing its accuracy. The division is usually done in a 70–30 ratio, with 70% of the data being used for training and 30% for testing. This process is repeated multiple times with varying size of chunks, using the benchmark data samples as inputs. This procedure is usually repeated 10 times to ensure accuracy.

The accuracy of each predictor is listed in Table 3. Similar to self‐consistency, RF has demonstrated outstanding results in independent tests compared to LGBM, ET, Bagging, Stacking, and XGB.

**TABLE 3:** Results of independent test.

	Acc	Sensitivity/Recall	Specificity	MCC
Random forest	91.44	92.59	90.17	82.83
LGBM	72.74	75.09	70.16	45.304
XGB	72.93	73.56	72.18	45.630
Extra trees	71.27	71.59	70.96	42.543
Bagging	71.09	75.52	66.01	41.748
Stacking	75.14	77.32	72.62	49.982

**FIGURE 5:**
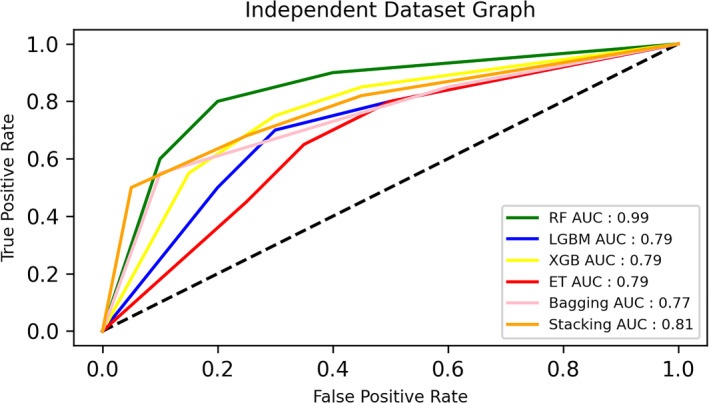
Independent test results of ROC.

Figure 5 illustrates a ROC curve of the Independent Test for various classifiers, specifically Random Forest, XGBoost, LGBM, Stacking, and Bagging, showcasing their respective performance in discriminating between classes.

### 4.3 Cross‐Validation

The validity of the results relies on comparing different models such as random forest, XGB, LGBM, Bagging, Extra Tree, and Stacking. Although its accuracy has not been assessed on new data, independent testing can give some insight into the performance of unseen data. However, it is not a foolproof method and may miss some significant parts during random selections or permutations. Therefore, cross‐validation is a more effective approach as it goes through all samples of the dataset by dividing it into non‐overlapping *k* folds and repeating the test *k* times. In each iteration, *k*−1 partitions are used for training and the average of the k‐tests indicates the predictor's accuracy. Cross‐validation is a reliable approach when test data is not available as no dataset is excluded during testing.

**FIGURE 6:**
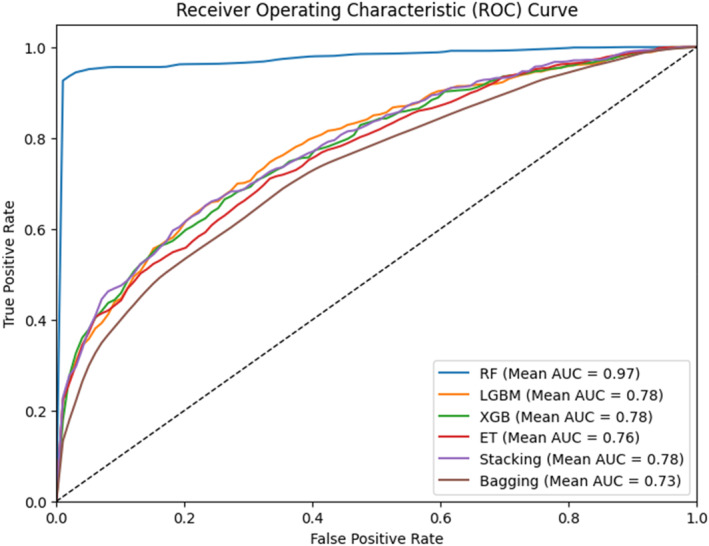
10‐fold ROC for PADG‐RF, PADG‐XGB, PADG‐ET, PADG‐LGBM, PADG‐Bagging & PADG stacking.

All predictors were evaluated using Ten‐Fold cross‐validation, where the dataset was divided into 10 folds and each fold was used for testing while the rest were used for training. Figure 6 illustrates 10‐fold ROC curves for various classifiers, specifically Random Forest, XGBoost, LGBM, Stacking, and Bagging, showcasing their respective performance in discriminating between classes.

The results show that RF has the highest accuracy level as shown in Table 4, which confirms the validity of the results as reported in previous observations.

**TABLE 4:** Cross‐validation ten‐fold results.

	Acc	Sensitivity/Recall	Specificity	MCC
Random forest	96.923	97.271	95.978	93.303
LGBM	70.643	72.435	70.034	41.410
XGB	70.315	72.984	70.103	40.748
Extra trees	68.102	69.364	66.794	36.312
Bagging	68.435	76.269	60.454	37.310
Stacking	73.284	72.295	70.289	40.061

A five‐fold test was also done for the same data samples to evaluate the predictor accuracy. Results are mentioned in Table 5 for all predictors (LGBM, XGB, Random forest, Extra Tree, Bagging & Stacking). Considering the average results populated fivefold it has been observed that RF is leading by 94.03% in accuracy.

**TABLE 5:** Cross‐validation 5 fold results.

	Acc	Sensitivity/Recall	Specificity	MCC
Random forest	94.03	94.532	93.518	88.060
LGBM	69.264	71.766	66.704	40.660
XGB	71.702	73.726	71.02	41.048
Extra trees	67.94	70.022	65.81	40.321
Bagging	67.94	74.726	61.006	36.081
Stacking	70.094	69.588	70.614	40.212

**FIGURE 7:**
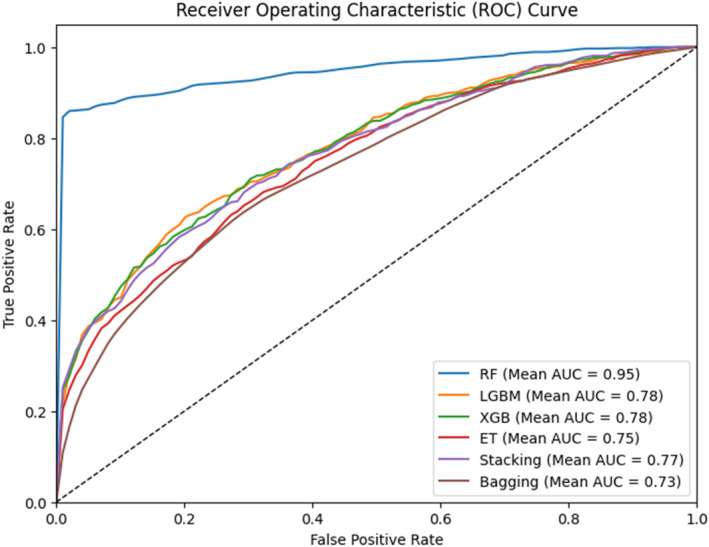
Five‐fold ROC for PADG‐RF, PADG‐XGB, PADG‐ET, PADG‐LGBM, PADG‐Bagging & PADG Stacking.

In the 5‐fold ROC analysis (Figure 7), various models demonstrated distinct performance in discriminating between positive and negative instances.

The early diagnosis of Parkinson's driver genes is crucial for timely treatment and therapy, highlighting the importance and demand for bioinformatics tools. The proposed computational predictor was developed through the systematic collection of benchmark datasets, the implementation of feature extraction, and model training with state‐of‐the‐art machine learning classifiers. The prediction model was then evaluated using various validation techniques, including independent testing, self‐consistency, 5‐fold cross‐validation, and 10‐fold cross‐validation. The results indicated that the PADG‐RF model demonstrated the best performance, with an accuracy rate of 92.27% in the independent validation method.

### 4.4 Boundary Graph

In this section, the effectiveness of classifiers is analysed through boundary and feature space visualisation comparisons. Decision boundary graphs are important because they provide a visual representation of how a machine learning model separates different classes in a dataset. This allows one to understand the model's behaviour, identify potential issues like overfitting or underfitting, and compare the performance of different models.

**FIGURE 8:**
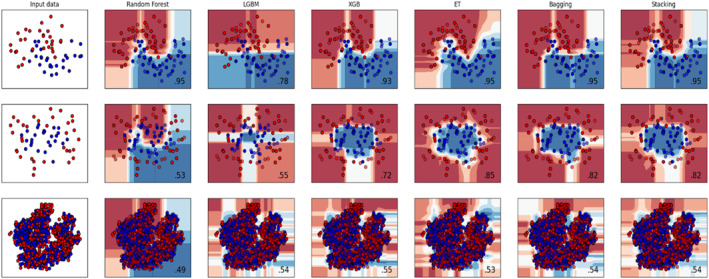
Boundary graph.

In Figure 8, it can be seen how different models (Random Forest, ET, Bagging, XGB, LGBM, Stacking) learn decision boundaries for increasingly complex datasets. The first row shows simple separation, the second row shows more complex patterns, and the third row shows highly intertwined classes. Some models, like XGB and Stacking, appear to adapt better to complex data, while others like LGBM struggle. This highlights the importance of choosing the right model for the task at hand. The t‐SNE maps high‐dimensional data (522 features) into a 2D space and may not yield distinct separations, especially with complex biological datasets such as Parkinson's gene expressions.

### 4.5 Feature Space Visualisation Comparison

**FIGURE 9:**
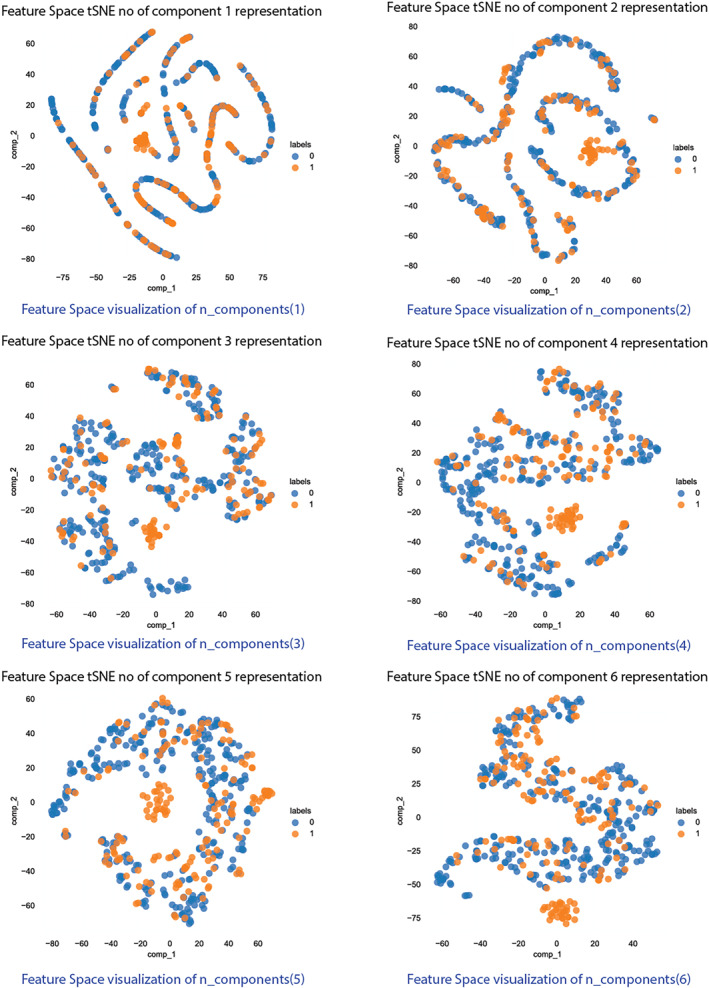
Feature space visualisation tSNE on n_components 1, 2, 3, 4, 5 & 6.

TSNE (t‐Distributed Stochastic Neighbour Embedding) is a dimensionality reduction technique used to visualise high‐dimensional data in 2D or 3D. This graph (Figure 9) shows how a dataset's structure changes as it is projected into fewer dimensions using t‐SNE. The first few components capture the most significant patterns, whereas later components might reveal finer details or noise.

As demonstrated, with deep representations, the boundary between positive and negative samples in the feature space is more distinctly defined, suggesting that the learning model will be able to distinguish between the classes with less effort.

## 5 Discussion

In our study, the process began with the selection of a benchmark dataset from the NCBI database, which provided comprehensive genetic information related to Parkinson's disease. Rigorous data processing techniques were then employed to ensure the dataset's integrity. This included removing irrelevant or incomplete sequences and applying normalisation and standardisation to make the data suitable for analysis.

Following the data cleaning phase, we applied sophisticated feature extraction methods to distill critical genetic insights. Techniques such as the PRIM (Position Relative Incidence Matrix), RPRIM (Reverse Position Relative Incidence Matrix), computation of FV (Frequency Vector), RAAPIV (Reverse Accumulative Absolute Position Incidence Vector) generation, and feature vector formulation were meticulously utilised. These methods adeptly captured complex genetic relationships and patterns, unveiling key markers linked to Parkinson's disease. The efficacy of these feature extraction techniques laid a robust foundation for evaluating classifier performance. By transforming raw genetic data into meaningful and interpretable feature vectors, we ensured that the classifiers had access to high‐quality inputs. This comprehensive approach facilitated accurate and reliable predictions, enhancing our understanding of genetic variations associated with Parkinson's disease and supporting the identification of crucial biomarkers.

The prediction performance of different classification models (random forest, bagging, complementary trees, LGBM, and stacking) was evaluated on the feature vectors retrieved to evaluate the genetic sequence. Random Forest demonstrates strong predictive power with an accuracy of 91.44%, making it a standout classifier in the evaluation of independent tests. Balanced sensitivity/recall scores (92.59) and specificity (90.17), as well as a high Matthews Correlation Coefficient (MCC) of 0.82838, further highlight its effectiveness. The power of RF in positive and negative prediction highlights the ability to effectively differentiate genetic data. In the analysed dataset, RF consistently outperformed other classifiers in cases. As an ensemble approach, RF combines multiple suggestions to minimise overfitting and exploit their complementary strengths, thereby improving prediction performance.

These findings align with the main goal of the study, which was to identify the most effective classifier for Parkinson's‐related genetic markers. They support the idea that ensemble methods, especially Random Forest, provide better accuracy and reliability than other classifiers. The strong accuracy, along with well‐balanced sensitivity and specificity, further confirms that Random Forest excels in handling complex genetic data, resulting in better performance than other models.

Despite some differences, all classifiers demonstrated promise in discerning genetic intricacies associated with Parkinson's, offering valuable insights for further study and diagnostic endeavours.

## 6 Conclusion

Timely identification of Parkinson's disease is essential for optimal treatment and support, as this neurodegenerative condition can severely affect individuals' lives. Genetic aspects also play a part in the progress of this debilitating disorder. Although there are several methods to diagnose Parkinson's which include voice, handwriting patterns, and image processing, their dependability remains a challenge. A prediction tool constructed on genetic sequences has been developed to progress initial and correct diagnosis. Parkinson's disease‐related biomarkers are identified from genetic profiles using advanced computational methods by this prediction tool. The study commenced with meticulous dataset preparation, followed by a multi‐faceted feature extraction phase. A range of mathematical techniques were applied, including the generation of PRIM and RPRIM matrices, frequency vector determination, and RAAPIV formulation. The resultant feature vectors served as the foundation for an extensive dataset training phase, employing a range of classification techniques. Ensemble models, such as Random Forest, boosting with XGBoost, and bagging were employed alongside individual classifiers like Extra Trees and LGBM. This multifaceted approach aimed to optimise the predictive accuracy of the model. The efficiency of classifiers is observed and calculated on dynamic hyperparameters to obtain an effective performance. Rigorous testing has been used for the predictor through various validation methods, to get accurate and reliable results. With the Random Forest predictor presenting the maximum accuracy rate in independent testing and cross‐validation, the results were promising. These results depicted that the PASD‐RF predictor attained remarkable accuracy rates of 91% in independent testing, 94% in 5‐fold, and 96% in 10‐fold cross‐validation. It was proved that the RF predictor is precise and suitable for diagnosing Parkinson's through genetic sequencing. By detecting associated biomarkers, this learning has the potential to enable timely diagnosis and treatment of Parkinson's Disease.

The approach is utilized among neurologists, researchers, and healthcare experts to detect Parkinson’s disease in its early stages. Moreover, it can be improved to support personalized treatment strategies by identifying specific genes associated with symptoms of Parkinson’s disease. Future advancements could focus on implementing the model with dynamic genomic profiles and extending its use to other neurodegenerative diseases, thereby enhancing diagnostic precision and improving patient care.

## Author Contributions

A.K. and Y.D.K. conceptualised the work and formulated a methodology to yield results. F.A. and T.A. analysed the data and Y.D.K. supervised the overall work. All authors contributed feedback and approved the final draft. All authors had final responsibility for the decision to submit for publication.

## Conflicts of Interest

The authors declare no conflicts of interest.

## Data Availability

The datasets generated during and/or analysed during the current study are available at GITHUB https://github.com/ashmeer/PADG‐Pred.

## References

[1] E. Arenas , “Parkinson’s Disease in the Single‐Cell Era,” Nature Neuroscience 25, no. 5 (2022): 536–538, 10.1038/s41593-022-01069-7. 35513514

[2] A. Hanikoğlu and E. Delen , “Biochemical Perspective on Parkinson’s Disease,” Multidisciplinary Approach in Medical Science III (2023).

[3] N. U. Islam , R. Khanam , and A. Kumar , “Using 3D CNN for Classification of Parkinson’s Disease From Resting‐State fMRI Data,” Journal of Engineering and Applied Science 70, no. 1 (2023): 89, 10.1186/s44147-023-00236-2.

[4] M. Abrishamdar , M. S. Jalali , and M. Rashno , “MALAT1 lncRNA and Parkinson’s Disease: The Role in the Pathophysiology and Significance for Diagnostic and Therapeutic Approaches,” Molecular Neurobiology 59, no. 9 (2022): 5253–5262, 10.1007/s12035-022-02899-z. 35665903

[5] A. Cherian , K. P. Divya , and A. Vijayaraghavan , “Parkinson's Disease–Genetic Cause,” Current Opinion in Neurology 36, no. 4 (2023): 292–301, 10.1097/wco.0000000000001167. 37366140

[6] S. Subramaniyan , B. B. Kuriakose , S. Mushfiq , N. Marimuthu Prabhu , and K. Muthusamy , “Gene Signals and SNPs Associated With Parkinson’s Disease: A Nutrigenomics and Computational Prospective Insights,” Neuroscience 533 (2023): 77–95, 10.1016/j.neuroscience.2023.10.007. 37858629

[7] G. Arena , K. Sharma , G. Agyeah , R. Krüger , A. Grünewald , and J. C. Fitzgerald , “Neurodegeneration and Neuroinflammation in Parkinson’s Disease: A Self‐Sustained Loop,” Current Neurology and Neuroscience Reports 22, no. 8 (2022): 427–440, 10.1007/s11910-022-01207-5. 35674870 PMC9174445

[8] C. Castillo‐Rangel , G. Marin , K. A. Hernández‐Contreras , et al., “Neuroinflammation in Parkinson’s Disease: From Gene to Clinic: A Systematic Review,” International Journal of Molecular Sciences 24, no. 6 (2023): 5792, 10.3390/ijms24065792. 36982866 PMC10051221

[9] T. M. Zohoncon , J. Sawadogo , A. Azaque Zoure , et al., “Gene Therapy for Parkinson’s Disease and Ethical Challenges: A Systematic Review,” Advances in Parkinson's Disease 12, no. 02 (2023): 9–28, 10.4236/apd.2023.122002.

[10] J. Cabana‐Domínguez , B. Torrico , A. Reif , N. Fernàndez‐Castillo , and B. Cormand , “Comprehensive Exploration of the Genetic Contribution of the Dopaminergic and Serotonergic Pathways to Psychiatric Disorders,” Translational Psychiatry 12 (2022): 1. 35013130 10.1038/s41398-021-01771-3 PMC8748838

[11] R. Formisano , K. D. Rosikon , A. Singh , and H. S. Dhillon , “The Dopamine Membrane Transporter Plays an Active Modulatory Role in Synaptic Dopamine Homeostasis,” Journal of Neuroscience Research 100, no. 8 (2022): 1551–1559, 10.1002/jnr.24965. 34747520 PMC9079189

[12] K. M. Costa and G. Schoenbaum , “Dopamine,” Current Biology 32, no. 15 (2022): 817–824, 10.1016/j.cub.2022.06.060. 35944478

[13] T. Behl , S. Kumar , Z. M. Althafar , et al., “Exploring the Role of Ubiquitin–Proteasome System in Parkinson's Disease,” Molecular Neurobiology 59, no. 7 (2022): 4257–4273, 10.1007/s12035-022-02851-1. 35505049

[14] Y. Li , S. Li , and H. Wu , “Ubiquitination‐proteasome System (UPS) and Autophagy Two Main Protein Degradation Machineries in Response to Cell Stress,” Cells 11, no. 5 (2022): 851, 10.3390/cells11050851. 35269473 PMC8909305

[15] H. J. Jang and K. C. Chung , “The Ubiquitin–Proteasome System and Autophagy Mutually Interact in Neurotoxin‐induced Dopaminergic Cell Death Models of Parkinson's Disease,” FEBS Letters 596, no. 22 (2022): 2898–2913, 10.1002/1873-3468.14479. 36054654

[16] M. Dehestani , H. Liu , A. A. Kumar Sreelatha , C. Schulte , V. Bansal , and T. Gasser , “Mitochondrial and Autophagy‐Lysosomal Pathway Polygenic Risk Scores Predict Parkinson's Disease,” Molecular and Cellular Neuroscience 121 (2022): 103751, 10.1016/j.mcn.2022.103751. 35710056

[17] S. Y.‐Y. Pang , R. C. N. Lo , P. Wing‐Lok Ho , et al., “LRRK2, GBA and Their Interaction in the Regulation of Autophagy: Implications on Therapeutics in Parkinson's Disease,” Translational Neurodegeneration 11 (2022): 1–14, 10.1186/s40035-022-00281-6. 35101134 PMC8805403

[18] H. Ye , L. A. Robak , M. Yu , M. Cykowski , and J. M. Shulman , “Genetics and Pathogenesis of Parkinson's Syndrome,” Annual Review of Pathology: Mechanisms of Disease 18, no. 1 (2023): 95–121, 10.1146/annurev-pathmechdis-031521-034145. PMC10290758 36100231

[19] A. H. Wise and R. N. Alcalay , “Genetics of Cognitive Dysfunction in Parkinson's Disease,” Progress in Brain Research (2022): 269. 10.1016/bs.pbr.2022.01.015 35248195

[20] K. Nishioka , Y. Imai , H. Yoshino , Y. Li , M. Funayama , and N. Hattori , “Clinical Manifestations and Molecular Backgrounds of Parkinson's Disease Regarding Genes Identified From Familial and Population Studies,” Frontiers in Neurology 13 (2022): 764917, 10.3389/fneur.2022.764917. 35720097 PMC9201061

[21] X. Kang , A. Ploner , Y. Wang , et al., “Genetic Overlap Between Parkinson’s Disease and Inflammatory Bowel Disease,” Brain Communications 5 (2023): 1, 10.1093/braincomms/fcad002. PMC9847552 36687396

[22] J. Lauritsen and M. Romero‐Ramos , “The Systemic Immune Response in Parkinson’s Disease: Focus on the Peripheral Immune Component,” Trends in Neurosciences 46, no. 10 (2023): 863–878, 10.1016/j.tins.2023.07.005. 37598092

[23] K. C. Paul , C. Kusters , M. Furlong , et al., “Immune System Disruptions Implicated in Whole Blood Epigenome‐Wide Association Study of Depression Among Parkinson's Disease Patients,” Brain, Behavior, & Immunity‐Health 26 (2022): 100530, 10.1016/j.bbih.2022.100530. PMC9618774 36325427

[24] P. N. Nalls Ma , C. M. Lill , C. B. Do , D. G. Hernandez , M. Saad , et al., “Large‐Scale Meta‐Analysis of Genome‐Wide Association Data Identifies Six New Risk Loci for Parkinson’s Disease,” Nature Genetics (2014): 489–993. 10.1038/ng.3043 PMC4146673 25064009

[25] J. Simon‐Sanchez , C. Paisan‐Ruiz , P. Lichtner , et al., “Genome‐Wide Association Study Reveals Genetic Risk Underlying Parkinson's Disease,” Nature Genetics 41, no. 12 (2009): 1308–1312, 10.1038/ng.487. 19915575 PMC2787725

[26] A. J. B. J. Noyce , L. Silveira‐Moriyama , H. R. Morris , N. Williams , D. J. Burn , et al., “Genome‐Wide Association Study of Parkinson's Disease in the Chinese Han Population,” Human Molecular Genetics (2009): 4472–4479.

[27] J. Quan , et al., “DaTscan SPECT Image Classification for Parkinson's Disease,” arXiv preprint arXiv (2019).

[28] H. R. KhachnaouiMabrouk and N. Khlifa , “Machine Learning and Deep Learning for Clinical Data and PET/SPECT Imaging in Parkinson’s Disease: A Review,” IET Image Processing 14, no. 16 (2020): 4013–4026, 10.1049/iet-ipr.2020.1048.

[29] M. Shaban , “Deep Convolutional Neural Network for Parkinson’s Disease Based Handwriting Screening,” in 2020 IEEE 17th International Symposium on Biomedical Imaging Workshops (ISBI Workshops), (IEEE, 2020), 1–4.

[30] C. R. Pereira , et al., “Convolutional Neural Networks Applied for Parkinson’s Disease Identification,” Machine Learning for Health Informatics: State‐of‐The‐Art and Future Challenges (2016): 377–390.

[31] A. Bourouhou , A. Jilbab , C. Nacir , and A. Hammouch , “Comparison of Classification Methods to Detect the Parkinson Disease,” in 2016 International Conference on Electrical and Information Technologies (ICEIT) (IEEE, 2016), 421–424.

[32] A. Khamparia , et al., “Sound Classification Using Convolutional Neural Network and Tensor Deep Stacking Network,” IEEE Access 7 (2019): 7717–7727, 10.1109/access.2018.2888882.

[33] E. Yu , R. Larivière , R. A. Thomas , et al., “Machine Learning Nominates the Inositol Pathway and Novel Genes in Parkinson’s Disease,” Brain 147, no. 3 (2024): 887–899, 10.1093/brain/awad345. 37804111 PMC10907089

[34] A. Ameli , L. Peña‐Castillo , and U. Hamid , “Assessing the Reproducibility of Machine‐Learning‐Based Biomarker Discovery in Parkinson’s Disease,” Biology and Medicine 174 (2024): 108407. 10.1016/j.compbiomed.2024.108407 38603902

[35] E. Glaab , “Computational Systems Biology Approaches for Parkinson’s Disease,” Cell and Tissue Research (2018): 373. 10.1007/s00441-017-2734-5 PMC6015628 29185073

[36] M. Pramanik , et al., “Machine Learning Methods With Decision Forests for Parkinson’s Detection,” Applied Sciences 11, no. 2 (2021): 581, 10.3390/app11020581.

[37] S. Kadiri , R. Kethireddy , and P. Alku , “Parkinson's Disease Detection From Speech Using Single Frequency Filtering Cepstral Coefficients,” in Interspeech (International Speech Communication Association (ISCA), 2020), 4971–4975.

[38] National Library of Medicine , (National Center for Biotechnology Information (NCBI), 1988).

[39] K. Yin , Xu Wen , S. Ren , et al., “Machine Learning Accelerates De Novo Design of Antimicrobial Peptides,” Interdisciplinary Sciences: Computational Life Sciences 16, no. 2 (2024): 1–12, 10.1007/s12539-024-00612-3. 38416364

[40] W. Alghamdi , M. E. M. Z. AlzahraniUllah , E. Alzahrani , M. Z. Ullah , and Y. D. Khan , “LBCEPred: A Machine Learning Model to Predict Linear B‐Cell Epitopes,” Briefings in Bioinformatics 23, no. 3 (2023): bbac035, 10.1093/bib/bbac035. 35262658

[41] S. Ahmed , M. Arif , M. Kabir , K. Khan , and Y. D. Khan , “PredAoDP: Accurate Identification of Antioxidant Proteins by Fusing Different Descriptors Based on Evolutionary Information With Support Vector Machine,” Chemometrics and Intelligent Laboratory Systems (2022): 228.

[42] S. Akbar and M. Hayat , “iMethyl‐STTNC, Identification of N6‐Methyladenosine Sites by Extending the Idea of SAAC into Chou's PseAAC to Formulate RNA Sequences,” Journal of Theoretical Biology 2018 (2018): 205–211, 10.1016/j.jtbi.2018.07.018. 30031793

[43] S. Ilyas , W. Hussain , A. Ashraf , Y. D. Khan , S. A. Khan , and K. C. Chou , “iMethylK‐PseAAC: Improving Accuracy of Lysine Methylation Sites Identification by Incorporating Statistical Moments and Position Relative Features into General PseAAC via Chou’s 5‐Steps Rule,” Current Genomics 20, no. 4 (2019): 275–292, 10.2174/1389202920666190809095206. 32030087 PMC6983956

[44] A. O. Almagrabi , Y. Daanial Khan , and S. Afzal Khan , “iPhosD‐PseAAC: Identification of Phosphoaspartate Sites in Proteins Using Statistical Moments and PseAAC,” Biocell 45, no. 5 (2021): 1287–1298, 10.32604/biocell.2021.013770.

[45] M. Awais , W. Hussain , Y. D. Khan , N. Rasool , S. A. Khan , and K. C. Chou , “iPhosH‐PseAAC: Identify Phosphohistidine Sites in Proteins by Blending Statistical Moments and Position Relative Features According to the Chou's 5‐step Rule and General Pseudo Amino Acid Composition,” IEEE/ACM Transactions on Computational Biology and Bioinformatics 18, no. 2 (2019): 596–610, 10.1109/tcbb.2019.2919025. 31144645

[46] S. A. Khan , Y. D. Khan , S. Ahmad , and K. H. Allehaibi , “N‐MyristoylG‐PseAAC: Sequence‐Based Prediction of N‐Myristoyl glycine Sites in Proteins by Integration of PseAAC and Statistical Moments,” Letters in Organic Chemistry 16, no. 3 (2019): 226–234, 10.2174/1570178616666181217153958.

[47] E. Alzahrani , W. Alghamdi , M. Z. Ullah , and Y. D. Khan , “Identification of Stress Response Proteins through Fusion of Machine Learning Models and Statistical Paradigms,” Scientific Reports 11 (2021): 1, 10.1038/s41598-021-99083-5. 34741132 PMC8571424

[48] Y. D. K. K Allehaibi and S. A. Khan , “ITAGPred: A Two‐Level Prediction Model for Identification of Angiogenesis and Tumor Angiogenesis Biomarker,” Applied Bionics and Biomechanics (2021): 2803147 34616486 10.1155/2021/2803147 PMC8490072

[49] A. Hassan , T. Alkhalifah , F. Alturise , and Y. D. Khan , “RCCC_Pred: A Novel Method for Sequence‐Based Identification of Renal Clear Cell Carcinoma Genes through DNA Mutations and a Blend of Features,” Diagnostics 12, no. 12 (2022): 3036, 10.3390/diagnostics12123036. 36553042 PMC9776995

[50] G. Perveen , F. T. Y. AlkhalifahDaanial Khan , T. Alkhalifah , and Y. Daanial Khan , “Hemolytic‐Pred: A Machine Learning‐Based Predictor for Hemolytic Proteins Using Position and Composition‐Based Features,” Digital Health 9 (2023): 9, 10.1177/20552076231180739. PMC10331097 37434723

[51] T. A. Mt Suleman , F. Alturise , and Y. D. Khan , “DHU‐Pred: Accurate Prediction of Dihydrouridine Sites Using Position and Composition Variant Features on Diverse Classifiers,” PeerJ 10 (2022): e14104, 10.7717/peerj.14104. 36320563 PMC9618264

[52] M. T. Suleman , F. Alturise , T. Alkhalifah , and Y. D. Khan , “iDHU‐Ensem: Identification of Dihydrouridine Sites Through Ensemble Learning Models,” Digital Health 9 (2023): 20552076231165963. 37009307 10.1177/20552076231165963 PMC10064468

[53] M. T. Suleman and Y. D. Khan , “PseU‐Pred: An Ensemble Model for Accurate Identification of Pseudouridine Sites,” Analytical Biochemistry (2023): 676. 10.1016/j.ab.2023.115247 37437648

[54] N. Alromema , M. T. Suleman , S. J. Malebary , A. Ahmed , B. A. M. Al‐Rami Al‐Ghamdi , and Y. D. Khan , “Identification of 6‐methyladenosine Sites Using Novel Feature Encoding Methods and Ensemble Models,” Scientific Reports 14, no. 1 (2024): 8180, 10.1038/s41598-024-58353-8. 38589431 PMC11001897

[55] M. T. Suleman and Y. Daanial Khan , “PseU‐Pred: An Ensemble Model for Accurate Identification of Pseudouridine Site,” Analytical Biochemistry (2023): 676. 10.1016/j.ab.2023.115247 37437648

[56] J. Chen , H. Liu , J. Yang , and K. C. Chou , “Prediction of Linear B‐Cell Epitopes Using Amino Acid Pair Antigenicity Scale,” Amino acids 33, no. 3 (2007): 423–428, 10.1007/s00726-006-0485-9. 17252308

[57] W. Chen , P. M. Feng , H. Lin , and K. C. Chou , “iRSpot‐PseDNC: Identify Recombination Spots With Pseudo Dinucleotide Composition,” Nucleic acids research 41, no. 6 (2013): 68, 10.1093/nar/gks1450. PMC3616736 23303794

